# Stalking Threat and Violent Behaviours in Erotomania: A Scoping Review of the Literature

**DOI:** 10.1192/bjo.2026.11222

**Published:** 2026-06-30

**Authors:** Corinne Calkam, Vaughan Bell, Alan Underwood, Kayla Gaw, Artemis Igoumenou

**Affiliations:** 1 University College London (UCL), London, United Kingdom; 2 Queen Mary University of London (QMUL), London, United Kingdom; 3 North London Forensic Service, London,United Kingdom; 4 Stalking Threat Assessment Centre (S-TAC), London, United Kingdom; 5 Centre for Addiction and Mental Health (CAMH), Toronto, Canada; 6 University of Toronto, Toronto, Canada

## Abstract

**Aims:**

Erotomanic delusions involve the fixed false belief that another person (often of higher social status) is in love with the individual. Although rare, erotomania is clinically significant due to its association with stalking, harassment, and, in some cases, serious violence. Risk assessment framework deficits have contributed to fragmented understanding of factors associated with violent escalation. Existing evidence is largely confined to case reports and case series, limiting systematic synthesis of clinical and contextual risk factors. This scoping review aimed to synthesise published case-level evidence on erotomania associated with stalking and/or violent behaviour. The primary objective was to identify individual, clinical, and contextual factors linked to stalking or violence in people with erotomanic delusions. Secondary objectives were to describe the nature and severity of violence and to identify gaps in the literature.

**Methods:**

A literature search was conducted in January 2026 using six databases. Eligible publications included case reports, case series, and observational or qualitative studies describing erotomanic delusions in connection with stalking or violence. Data were extracted using Covidence capturing sociodemographic, clinical and criminological factors. Results were synthesised descriptively and reported as frequencies and percentages overall and by sex. Fifty publications comprising 87 cases were included.

**Results:**

The sample included 51 males (58.6%) and 36 females (41.4%), with a mean age of 36.0 years (SD 11.7). Most individuals were single, socially isolated and had unstable living arrangements. Erotomanic delusions were most commonly described in the context of a primary diagnosis of a psychotic illness (90.8%). Co-occurring delusional themes were common, particularly referential, persecutory, and grandiose delusions.

Stalking was reported in 88.5% of cases, typically involving a single object. Victims were predominantly female (64.5%) and most often professionals (e.g. clinicians, employers). Violence occurred in 49.4% of cases, with males significantly more likely to perpetrate severe violence. Common motivations included delusional beliefs of reciprocated love and perceived rejection. Criminal charges arose in over half of cases, particularly among male offenders.

**Conclusion:**

Erotomania associated with stalking and violence represents a high-risk clinical and forensic phenomenon. The literature remains dominated by descriptive reports underscoring the need for structured risk assessment approaches, clearer diagnostic guidance, and targeted management strategies to prevent escalation and harm.

